# Synthesis and biological evaluation of anti-*Toxoplasma gondii* activity of a novel scaffold of thiazolidinone derivatives

**DOI:** 10.1080/14756366.2017.1316494

**Published:** 2017-05-24

**Authors:** Simone Carradori, Daniela Secci, Bruna Bizzarri, Paola Chimenti, Celeste De Monte, Paolo Guglielmi, Cristina Campestre, Daniela Rivanera, Claudia Bordón, Lorraine Jones-Brando

**Affiliations:** a Department of Pharmacy, “G. D’Annunzio” University of Chieti-Pescara, Chieti, Italy;; b Department of Drug Chemistry and Technologies, Sapienza University of Rome, Rome, Italy;; c Department of Public Health and Infectious Diseases, Sapienza University of Rome, Rome, Italy;; d Stanley Division of Developmental Neurovirology, Johns Hopkins University School of Medicine, Baltimore, MD, USA

**Keywords:** Toxoplasma, parasite growth inhibition, host cell invasion, cytotoxicity, 1,3-thiazolidin-4-one, ferrocene

## Abstract

We designed and synthesised novel *N*-substituted 1,3-thiazolidin-4-one derivatives for the evaluation of their anti-*Toxoplasma gondii* efficacy. This scaffold was functionalised both at the N1-hydrazine portion with three structurally different moieties and at the lactam nitrogen with substituted benzyl groups selected on the basis of our previous structure-activity relationships studies. Using three different assay methods, the compounds were assessed *in vitro* to determine both the levels of efficacy against the tachyzoites of *T. gondii* (IC_50_ = 5–148 μM), as well as any evidence of cytotoxicity towards human host cells (TD_50_ = 68 to ≥320 μM). Results revealed that ferrocene-based thiazolidinones can possess potent anti-tachyzoite activity (TI =2–64).

## 1. Introduction

*Toxoplasma gondii* is the eukaryotic pathogen responsible for toxoplasmosis, a major parasitic disease of global importance that afflicts both humans and animals^1^^,^^2^. *Toxoplasma gondii* is an obligate intracellular parasite endowed with a complex life cycle during which the parasite has the ability to differentiate from the rapidly replicating form (tachyzoite) to the metabolically less active form (bradyzoite) that is enclosed in a tissue cyst, and *vice versa*. Tachyzoites are responsible for establishing an acute infection in humans and other animals *via* ingestion of undercooked meat carrying parasite tissue cysts, by consumption of foods or water contaminated with parasite oocysts disseminated by infected felids, or *via* transplacental passage in pregnant women^3–5^. Formation of bradyzoite-bearing tissue cysts in the host signals establishment of the chronic, often quiescent, form of disease. Immune suppression on the backdrop of such chronic toxoplasmosis can lead to reactivation of acute toxoplasmosis, potentially resulting in further serious diseases including encephalitis^6^, development of schizophrenia^7^^,^^8^, spontaneous abortions in pregnant women, and ocular diseases^9^.

Toxoplasmosis is currently treated with compounds that have been associated with severe side effects ranging from intolerance to allergic reactions^10^^,^^11^. Additionally, these drugs are unable to completely get rid of the host of parasite tissue cysts, and thus cannot cure the chronic infection^12^ leaving infected individuals, especially immunocompromised hosts, susceptible to serious sequelae. Many novel and often highly efficacious *Toxoplasma* inhibitors have been recently reported, yet complete eradication of bradyzoite cysts remains elusive^13–17^. Our objective is to develop a compound that is highly efficacious against both the tachyzoites and the bradyzoites of *T. gondii* with minimal host cell cytotoxicity. We report here our continuing efforts towards this objective beginning with a focus on anti-tachyzoite activity. Pursuing our research on the thiazole derivatives endowed with anti-*Toxoplasma* activity and limited cytotoxicity^18^, we recently published a new series of thiazolidinones substituted or not with a benzyl group at the lactam NH^19^ exploring which substituents at the N1-hydrazine portion of the lateral chain presented either a promising anti-*Toxoplasma* activity in the micromolar range and a better ability to inhibit the penetration of parasite into the host cell. Starting from these results, we synthesised 33 thiazolidinone derivatives and tested them for *in vitro* anti-parasitic activity using *T. gondii* tachyzoites. The newly synthesised compounds, with respect to the previous series, kept the thiazolidinone pharmacophore constant varying the chemical space at the nitrogen atoms. After spectroscopic characterisation to ensure purity, the newly synthesised compounds were evaluated for parasite growth inhibition and cytotoxicity, inhibition of tachyzoite invasion of host cells, and inhibition of intracellular tachyzoite replication.

## 2. Chemistry

**Scheme 1.**
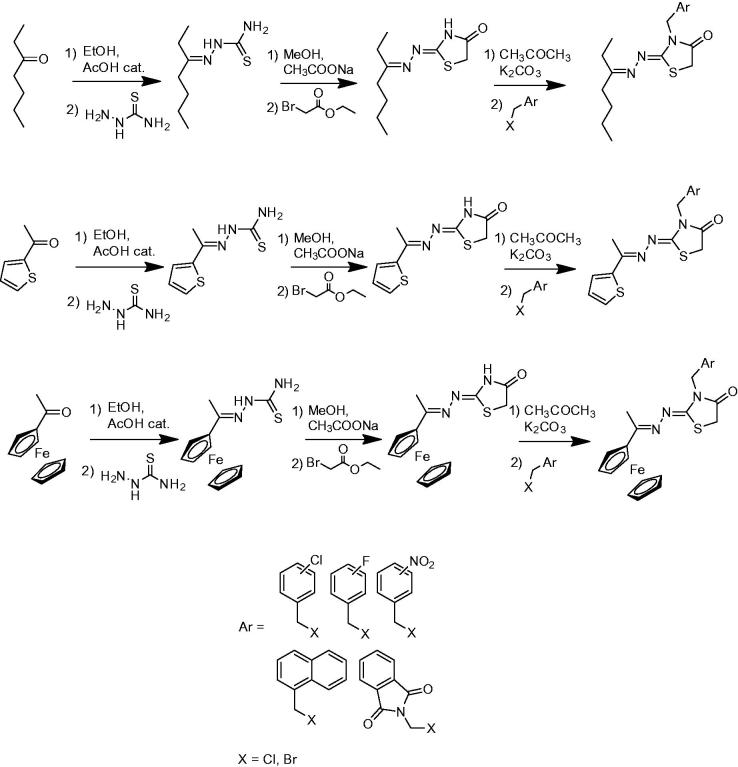
General synthesis of the derivatives **1**–**33**.

As outlined in Scheme 1, for the synthesis of compounds **1–33** three different carbonyl compounds (3-heptanone, 2-acetylthiophene, and acetylferrocene) were dissolved/suspended in ethyl alcohol and reacted directly with thiosemicarbazide and catalytic amounts of acetic acid. The resulting intermediate thiosemicarbazones were reacted with ethyl bromoacetate in methanol and sodium acetate in order to obtain the corresponding 1,3-thiazolidin-4-one derivatives^20^. Finally, the reaction between the resulting products and ortho-, meta-, or para-substituted (with nitro groups and halogens) benzyl bromides or 1-(chloromethyl)naphthalene or *N*-(chloromethyl)phthalimide in anhydrous acetone and potassium carbonate gave the *N*-benzyl derivatives (**1–33**). All the synthesised compounds were washed with petroleum ether and diethyl ether and purified by column chromatography before characterisation by spectroscopic methods (IR and ^1 ^H/^13 ^C NMR) and elemental analysis. Some representative spectra are reported in the Supplementary materials.

## 3. Biological characterisation

**Figure 1.**
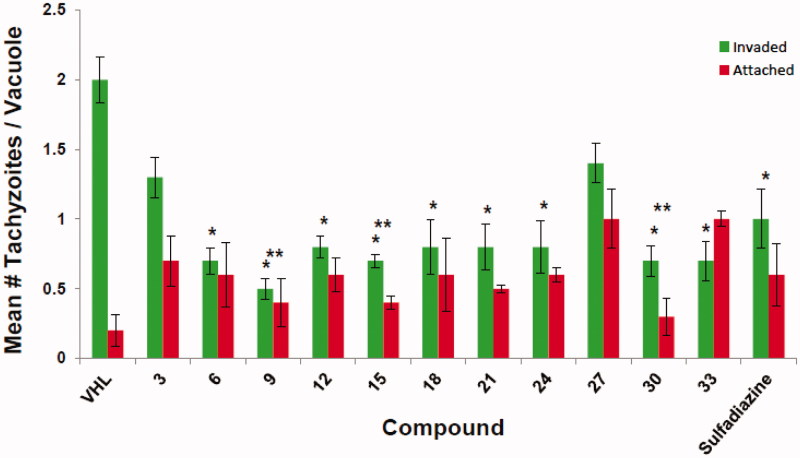
Invasion assay of ferrocene-based thiazolidinone derivatives (10 μM). VHL: vehicle (DMSO). *Significant inhibition of tachyzoite invasion (*p* ≤ .05, two-tailed Students’ *t* test). **Significant inhibition of tachyzoite attachment (*p* ≤ .05, two-tailed Students’ *t* test). Data are compiled from results of three independent experiments.

**Figure 2.**
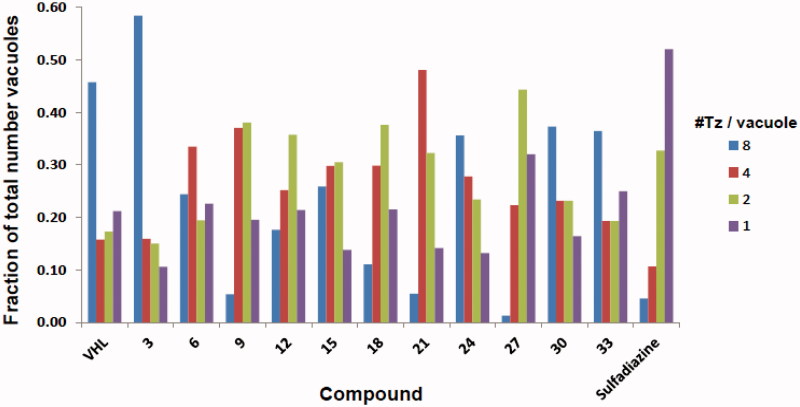
Replication assay of ferrocene-based thiazolidinone derivatives (10 μM). VHL: vehicle (DMSO); Tz: tachyzoites. Data are compiled from results of three independent experiments.

Using published methods^8^^,^^19^, all of the prepared *N*-substituted thiazolidinones derivatives were evaluated *in vitro* for (i) host cell cytotoxicity as well as for the ability to inhibit tachyzoite growth over a period of 5 days (Table 1), for (ii) the ability to inhibit tachyzoite invasion of host cells (Figure 1), and for (iii) the ability to inhibit intracellular tachyzoite replication (Figure 2).

**Table 1.** Five-day growth inhibition assay for *N*-substituted thiazolidinone derivatives **1**–**33** and reference drugs.

Compound	Structure	IC_50_^a^ μM	IC_90_^b^ μM	TD_50_^c^ μM	TI^d^
**1**		20	111	236	12
**2**		37	155	280	8
**3**		15	78	≥320	21
**4**		44	138	68	2
**5**		52	171	≥320	6
**6**		24	152	≥320	13
**7**		32	128	98	3
**8**		148	378	≥320	2
**9**		73	292	217	3
**10**		24	104	90	4
**11**		25	103	82	3
**12**		17	64	≥320	19
**13**		18	99	246	14
**14**		45	147	178	4
**15**		8	57	≥320	40
**16**		29	107	192	7
**17**		72	312	≥320	4
**18**		6	16	≥320	53
**19**		39	183	95	2
**20**		34	112	84	2
**21**		9	24	≥320	36
**22**		19	106	165	9
**23**		51	256	147	3
**24**		8	17	≥320	40
**25**		51	131	244	5
**26**		81	211	≥320	4
**27**		10	55	≥320	32
**28**		37	138	271	7
**29**		35	122	≥320	9
**30**		5	17	≥320	64
**31**		88	402	≥320	4
**32**		78	219	117	2
**33**		82	286	138	2
Sulfadiazine		43	219	281	7
Atovaquone		0.2	2	24	133

a IC_50_= Median inhibitory concentration, a measure of tachyzoite inhibition.

b IC_90_= Concentration at which 90% of the tachyzoite growth is inhibited.

c TD_50_= Median toxicity dose, a measure of cytotoxicity against host cells.

d TI = Therapeutic index, a measure of efficacy, calculated by TD_50_/IC_50_. When TD_50_ ≥320 TI = 320/IC_50_.

## 4. Experimental protocols

The chemicals, solvents for synthesis, and spectral grade solvents were purchased from Aldrich (Italy) and used without further purification. Melting points (uncorrected) were determined automatically on an FP62 apparatus (Mettler-Toledo, Colombus, OH). Neat IR spectra were registered on a Perkin Elmer FT-IR Spectrometer Spectrum 1000. ^1 ^H (and ^13^C) NMR spectra were recorded at 400 (and 101) MHz on a Bruker spectrometer using CDCl_3_ or DMSO-d_6_ as solvent. Chemical shifts are expressed as *δ* units (parts per millions) relative to the solvent peak. Coupling constants *J* are valued in Hertz (Hz). Elemental analyses for C, H, and N were recorded on a Perkin-Elmer 240 B microanalyzer and the analytical results were within ±0.4% of the theoretical values for all compounds. All reactions were monitored by TLC performed on 0.2 mm thick silica gel plates (60 F_254_ Merck). In general, the IR spectrum for derivatives **1–33** showed bands at about 3027 cm^−1^ (C_sp2_-H stretching), at about 1690 (C = O stretching), at about 1620 (C = N stretching), and at about 1580 and 1440 (C = C stretching). Some representative spectra were reported in the Supplementary materials.

### 4.1. General procedure for the synthesis of compounds 1–33

The initial carbonyl compound (50 mmol) was dissolved/suspended in ethanol (50 ml) and magnetically stirred with thiosemicarbazide (50 mmol) and catalytic amounts of acetic acid for 8–24 h at room temperature. The obtained thiosemicarbazone was filtered, washed with appropriate solvent (*n*-hexane, petroleum ether, or diethyl ether) and dried under vacuum overnight. The intermediate thiosemicarbazone (50 mmol) reacted with ethyl bromoacetate (50 mmol), in methanol (50 ml) and sodium acetate (50 mmol) at room temperature under magnetic stirring for 24 h. The resulting 4-thiazolidinone was poured on ice, filtered or extracted with chloroform (3 × 30 ml) and purified by column chromatography (SiO_2_, ethyl acetate/*n*-hexane). Then, the resulting thiazolidinone (50 mmol) was dissolved/suspended in 50 ml of anhydrous acetone in the presence of anhydrous potassium carbonate (50 mmol), and reacted with equimolar amounts of 2-,3-,4-nitrobenzyl bromide, 2-,3-,4-chlorobenzyl bromide, 2-,3-,4-fluorobenzyl bromide, 1-(chloromethyl)naphthalene or *N*-(chloromethyl)phthalimide for 24–48 h. The products were poured on ice, filtered or extracted with chloroform (3 × 50 ml) and purified by column chromatography (SiO_2_, ethyl acetate/*n*-hexane) in order to obtain the title compounds in high yields as previously reported^20^.

#### 4.1.1. 2-((Heptan-3-ylidene)hydrazono)-3-(2-nitrobenzyl)thiazolidin-4-one (1)

White powder, mp 67–69 °C, 95% yield; ^1^H NMR (400 MHz, DMSO-d_6_): *δ* 0.66–0.69 (m, 3H, CH_3_), 0.85–0.88 (m, 3H, CH_3_), 1.26–1.29 (m, 2H, CH_2_), 1.41–1.46 (m, 2H, CH_2_), 2.07–2.09 (m, 2H, CH_2_), 2.18–2.22 (m, 2H, CH_2_), 4.04 (s, 2H, CH_2_, thiazolidinone), 5.13 (s, 2H, ArCH_2_), 7.36–7.40 (m, 1 H, Ar), 7.56–7.58 (m, 1 H, Ar), 7.68–7.74 (m, 1 H, Ar), 8.06–8.08 (m, 1 H, Ar). ^13^C NMR (101 MHz, DMSO-d_6_) *δ* 10.9 (CH_3_), 14.3 (CH_3_), 22.3 (CH_2_), 28.1 (CH_2_), 29.7 (CH_2_), 32.6 (CH_2_), 35.6 (CH_2_, thiazolidinone), 42.9 (CH_2_), 125.2 (Ar), 128.6 (Ar), 129.1 (Ar), 131.4 (Ar), 134.4 (Ar), 148.7 (Ar), 158.3 (C = N, thiazolidinone), 159.6 (C = N), 172.7 (C = O). Anal. Calcd for C_17_H_22_N_4_O_3_S: C, 56.33; H, 6.12; N, 15.46. Found: C, 56.67; H, 6.31; N, 15.20.

#### 4.1.2. 3-(2-Nitrobenzyl)-2-((1-(thiophen-2-yl)ethylidene)hydrazono)thiazolidin-4-one (2)

Pink powder, mp 150–151 °C, 93% yield; ^1 ^H NMR (400 MHz, CDCl_3_): *δ* 2.26 (s, 3 H, CH_3_), 3.90 (s, 2 H, CH_2_, thiazolidinone), 5.44 (s, 2 H, ArCH_2_), 7.05–7.06 (m, 1 H, thiophene), 7.32–7.48 (m, 4 H, thiophene + Ar), 7.59–7.61 (m, 1 H, Ar), 8.07–8.09 (m, 1 H, Ar). Anal. Calcd for C_16_H_14_N_4_O_3_S_2_: C, 51.32; H, 3.77; N, 14.96. Found C, 51.65; H, 3.50; N, 15.11.

#### 4.1.3. 3-(2-Nitrobenzyl)-2-((1-(ferrocen-2-yl)ethylidene)hydrazono)thiazolidin-4-one (3)

Red powder, mp 195–198 °C, 90% yield; ^1^H NMR (400 MHz, DMSO-d_6_): *δ* 2.02 (s, 3H, CH_3_), 4.05 (s, 2H, CH_2_, thiazolidinone), 4.15–4.17 (m, 5H, ferrocene), 4.41 (s, 2H, ferrocene), 4.65 (s, 2H, ferrocene), 5.21 (s, 2H, ArCH_2_), 7.46–7.47 (t, 1H, Ar), 7.58–7.59 (m, 1H, Ar), 7.73–7.75 (m, 1H, Ar), 8.08–8.10 (d, *J* = 7.2 Hz, 1 H, Ar). ^13^C NMR (101 MHz, DMSO-d_6_) *δ* 15.8 (CH_3_), 32.7 (CH_2_, thiazolidinone), 42.9 (CH_2_), 67.8 (CH_2_, ferrocene), 69.7 (CH_2_, ferrocene), 70.6 (CH, ferrocene), 82.8 (C, ferrocene), 125.2 (Ar), 129.0 (Ar), 129.2 (Ar) 131.3 (Ar), 134.5 (Ar), 148.8 (Ar), 159.2 (C = N, thiazolidinone), 164.6 (C = N), 172.8 (C = O). Anal. Calcd for C_22_H_20_FeN_4_O_3_S: C, 55.47; H, 4.23; N, 11.76. Found: C, 55.23; H, 4.45; N, 11.98.

#### 4.1.4. 2-((Heptan-3-ylidene)hydrazono)-3–(3-nitrobenzyl)thiazolidin-4-one (4)

Yellow oil, 65% yield; ^1^H NMR (400 MHz, DMSO-d_6_): *δ* 0.64–0.69 (m, 6H, 2 × CH_3_), 0.82–0.84 (m, 1H, CH_2_), 1.09–1.11 (m, 2H, CH_2_), 1.26–1.28 (m, 1H, CH_2_), 2.02–2.12 (m, 4H, 2 × CH_2_), 3.79 (s, 2H, CH_2_, thiazolidinone), 4.77 (s, 2H, ArCH_2_), 7.42–7.47 (m, 1H, Ar), 7.54–7.59 (m, 1H, Ar), 7.94–7.95 (m, 2H, Ar). ^13^C NMR (101 MHz, DMSO-d_6_) *δ* 11.0 (CH_3_), 14.0 (CH_3_), 22.7 (CH_2_), 28.5 (CH_2_), 29.6 (CH_2_), 30.8 (CH_2_), 32.5 (CH_2_, thiazolidinone), 45.6 (CH_2_), 122.9 (Ar), 123.0 (Ar), 130.4 (Ar), 134.7 (Ar), 139.0 (Ar), 148.2 (Ar), 158.9 (C = N, thiazolidinone), 160.4 (C = N), 172.7 (C = O). Anal. Calcd for C_17_H_22_N_4_O_3_S: C, 56.33; H, 6.12; N, 15.46. Found: C, 56.59; H, 5.94; N, 15.25.

#### 4.1.5. 3-(3-Nitrobenzyl)-2-((1-(thiophen-2-yl)ethylidene)hydrazono)thiazolidin-4-one (5)

Yellow powder, mp 165–170 °C, 82% yield; ^1^H NMR (400 MHz, DMSO-d_6_): *δ* 2.36 (s, 3H, CH_3_), 4.05 (s, 2H, CH_2_, thiazolidinone), 5.05 (s, 2H, ArCH_2_), 7.11–7.13 (m, 1H, thiophene), 7.54–7.55 (m, 1H, thiophene), 7.61–7.62 (m, 1H, thiophene), 7.65–7.69 (m, 1H, Ar), 7.83–7.85 (m, 1H, Ar), 8.17–8.19 (m, 1H, Ar), 8.30 (s, 1H, Ar). Anal. Calcd for C_16_H_14_N_4_O_3_S_2_: C, 51.32; H, 3.77; N, 14.96. Found: C, 51.19; H, 3.95; N, 15.16.

#### 4.1.6. 3-(3-Nitrobenzyl)-2-((1-(ferrocen-2-yl)ethylidene)hydrazono)thiazolidin-4-one (6)

Red powder, mp 171–173 °C, 83% yield; ^1^H NMR (400 MHz, DMSO-d_6_): *δ* 2.22 (s, 3H, CH_3_), 4.01 (s, 2H, CH_2_, thiazolidinone), 4.17 (s, 5H, ferrocene), 4.43 (s, 2H, ferrocene), 4.69 (s, 2H, ferrocene), 5.03 (s, 2H, ArCH_2_), 7.66–7.70 (*t*, 1H, Ar), 7.84–7.86 (d, *J* = 7.2 Hz, 1H, Ar), 8.18–8.19 (d, *J* = 7.2 Hz, 1H, Ar), 8.32 (s, 1H, Ar). ^13^C NMR (101 MHz, DMSO-d*_6_*) *δ* 16.2 (CH_3_), 32.5 (CH_2_, thiazolidinone), 45.6 (CH_2_), 67.8 (CH_2_, ferrocene), 69.7 (CH_2_, ferrocene), 70.6 (CH, ferrocene), 82.9 (C, ferrocene), 123.1 (Ar), 123.7 (Ar), 130.5 (Ar), 135.5 (Ar), 138.8 (Ar), 148.2 (Ar), 159.8 (C = N, thiazolidinone), 164.6 (C = N), 172.8 (C = O). Anal. Calcd for C_22_H_20_FeN_4_O_3_S: C, 55.47; H, 4.23; N, 11.76. Found: C, 55.68; H, 4.11; N, 11.57.

#### 4.1.7. 2-((Heptan-3-ylidene)hydrazono)-3-(4-nitrobenzyl)thiazolidin-4-one (7)

Yellow oil, 63% yield; ^1^H NMR (400 MHz, CDCl_3_): *δ* 0.86–0.89 (t, 3H, CH_3_), 0.93–1.01 (m, 2H, CH_2_), 1.13–1.18 (m, 1H, CH_2_), 1.22–1.31 (m, 2H, CH_2_), 1.35–1.42 (m, 1H, CH_2_), 1.54–1.62 (m, 1H, CH_2_), 2.31–2.43 (m, 1H, CH_2_, 3H, CH_3_), 3.81 (s, 2H, CH_2_, thiazolidinone), 5.07 (s, 2H, ArCH_2_), 7.56–7.61 (m, 2H, Ar), 8.18–8.20 (d, *J* = 8.8 Hz, 2H, Ar). ^13^C NMR (101 MHz, DMSO-d*_6_*) *δ* 11.0 (CH_3_), 14.0 (CH_3_), 22.8 (CH_2_), 28.6 (CH_2_), 29.6 (CH_2_), 30.9 (CH_2_), 32.5 (CH_2_, thiazolidinone), 45.7 (CH_2_), 124.0 (Ar), 128.7 (Ar), 128.8 (Ar), 129.2 (Ar), 144.6 (Ar), 147.2 (Ar), 158.7 (C = N, thiazolidinone), 160.1 (C = N), 172.6 (C = O). Anal. Calcd for C_17_H_22_N_4_O_3_S: C, 56.33; H, 6.12; N, 15.46. Found: C, 56.19; H, 6.27; N, 15.68.

#### 4.1.8. 3-(4-Nitrobenzyl)-2-((1-(ferrocen-2-yl)ethylidene)hydrazono)thiazolidin-4-one (9)

Red powder, mp 214–217 °C, 84% yield; ^1^H NMR (400 MHz, DMSO-d_6_): *δ* 2.15 (s, 3H, CH_3_), 4.03 (s, 2H, CH_2_, thiazolidinone), 4.17 (s, 5H, ferrocene), 4.42 (s, 2H, ferrocene), 4.67 (s, 2H, ferrocene), 5.04 (s, 2H, ArCH_2_), 7.64–7.66 (d, *J* = 8.8 Hz, 2 H, Ar), 8.23–8.25 (d, *J* = 8.8 Hz, 2H, Ar). ^13^C NMR (101 MHz, DMSO-d_6_) *δ* 16.2 (CH_3_), 32.6 (CH_2_, thiazolidinone), 45.8 (CH_2_), 67.8 (CH_2_, ferrocene), 69.7 (CH_2_, ferrocene), 70.6 (CH, ferrocene), 82.9 (C, ferrocene), 124.0 (Ar), 129.4 (Ar), 144.4 (Ar), 147.4 (Ar), 159.5 (C = N, thiazolidinone), 164.6 (C = N), 172.7 (C = O). Anal. Calcd for C_22_H_20_FeN_4_O_3_S: C, 55.47; H, 4.23; N, 11.76. Found: C, 55.68; H, 4.11; N, 11.57.

#### 4.1.9. 3-(2-Fluorobenzyl)-2-((heptan-3-ylidene)hydrazono)thiazolidin-4-one (10)

Yellow oil, 64% yield; ^1^H NMR (400 MHz, DMSO-d_6_): *δ* 0.75–0.80 (m, 3H, CH_3_), 0.85–0.90 (m, 3H, CH_3_), 1.27–1.32 (m, 2H, CH_2_), 1.42–1.48 (m, 2H, CH_2_), 2.21–2.26 (m, 4H, 2 × CH_2_), 4.00 (s, 2H, CH_2_, thiazolidinone), 4.91 (s, 2H, ArCH_2_), 7.12–7.35 (m, 4H, Ar). ^13^C NMR (101 MHz, DMSO-d_6_) *δ* 11.0 (CH_3_), 14.1 (CH_3_), 22.7 (CH_2_), 28.5 (CH_2_), 29.6 (CH_2_), 30.8 (CH_2_), 32.4 (CH_2_, thiazolidinone), 35.6 (CH_2_), 115.7 (Ar), 123.4 (Ar), 124.8 (Ar), 129.4 (Ar), 129.8 (Ar), 159.2 (C = N, thiazolidinone), 160.1 (C = N), 161.7 (Ar, C-F), 172.5 (C = O). Anal. Calcd for C_17_H_22_FN_3_OS: C, 60.87; H, 6.61; N, 12.53. Found: C, 60.54; H, 6.39; N, 12.68.

#### 4.1.10. 3-(2-Fluorobenzyl)-2-((1-(thiophen-2-yl)ethylidene)hydrazono)thiazolidin-4-one (11)

Orange powder, mp 80–85 °C, 91% yield; ^1^H NMR (400 MHz, DMSO-d_6_): *δ* 2.28 (s, 3H, CH_3_), 4.06 (s, 2H, CH_2_, thiazolidinone), 4.98 (s, 2H, ArCH_2_), 7.10–7.21 (m, 3H, thiophene), 7.33–7.36 (m, 2H, Ar), 7.51–7.52 (m, 1H, Ar), 7.61–7.62 (m, 1H, Ar). Anal. Calcd for C_16_H_14_FN_3_OS_2_: C, 55.31; H, 4.06; N, 12.09. Found: C, 55.60; H, 3.83; N, 12.21.

#### 4.1.11. 3-(2-Fluorobenzyl)-2-((1-(ferrocen-2-yl)ethylidene)hydrazono)thiazolidin-4-one (12)

Red oil, 65% yield; ^1^H NMR (400 MHz, DMSO-d_6_): *δ* 2.14 (s, 3H, CH_3_), 4.02 (s, 2H, CH_2_, thiazolidinone), 4.16 (s, 5H, ferrocene), 4.42 (s, 2H, ferrocene), 4.67 (s, 2H, ferrocene), 4.96 (s, 2H, ArCH_2_), 7.19–7.24 (m, 2H, Ar), 7.33–7.38 (m, 2H, Ar). ^13^C NMR (101 MHz, DMSO-d_6_) *δ* 16.0 (CH_3_), 32.4 (CH_2_, thiazolidinone), 67.8 (CH_2_, ferrocene), 69.7 (CH_2_, ferrocene), 70.5 (CH, ferrocene), 82.9 (C, ferrocene), 115.7 (Ar), 123.4 (Ar), 124.8 (Ar), 129.9 (Ar), 130.1 (Ar), 159.4 (C = N, thiazolidinone), 161.8 (Ar, C–F), 164.4 (C = N), 172.5 (C = O). Anal. Calcd for C_22_H_20_FFeN_3_OS: C, 58.81; H, 4.49; N, 9.35. Found: C, 58.69; H, 4.22; N, 9.07.

#### 4.1.12. 3-(3-Fluorobenzyl)-2-((heptan-3-ylidene)hydrazono)thiazolidin-4-one (13)

Yellow powder, mp 30–31 °C, 81% yield; ^1 ^H NMR (400 MHz, DMSO-d_6_): *δ* 0.55–0.59 (m, 1 H, CH_2_), 0.62–0.70 (m, 3 H, CH_3_), 0.81–0.85 (m, 1 H, CH_2_), 0.92–1.12 (m, 4 H, 2 × CH_2_), 1.27–1.31 (m, 1 H, CH_2_), 2.03–2.12 (m, 4 H, CH_2_ + CH_3_), 3.79 (s, 2 H, CH_2_, thiazolidinone), 4.66 (s, 2 H, ArCH_2_), 6.88–6.96 (m, 3 H, Ar), 7.15–7.23 (m, 1 H, Ar). ^13^C NMR (101 MHz, DMSO-d_6_) *δ* 11.0 (CH_3_), 14.3 (CH_3_), 22.3 (CH_2_), 28.2 (CH_2_), 29.6 (CH_2_), 30.9 (CH_2_), 32.4 (CH_2_, thiazolidinone), 45.7 (CH_2_), 115.2 (Ar), 123.9 (Ar), 124.2 (Ar), 130.8 (Ar), 139.5 (Ar), 160.0 (C = N, thiazolidinone), 160.4 (C = N), 162.5 (d, *J_C–F_* = 244.4 Hz, Ar, C–F), 172.6 (C = O). Anal. Calcd for C_17_H_22_FN_3_OS: C, 60.87; H, 6.61; N, 12.53. Found: C, 60.61; H, 6.82; N, 12.36.

#### 4.1.13. 3-(3-Fluorobenzyl)-2-((1-(thiophen-2-yl)ethylidene)hydrazono)thiazolidin-4-one (14)

Yellow powder, mp 84–85 °C, 89% yield; ^1 ^H NMR (400 MHz, DMSO-d_6_): *δ* 2.33 (s, 3 H, CH_3_), 4.05 (s, 2 H, CH_2_, thiazolidinone), 4.93 (s, 2 H, ArCH_2_), 7.12–7.22 (m, 4 H, thiophene + Ar), 7.38–7.41 (m, 1 H, Ar), 7.53 (bs, 1 H, Ar), 7.61–7.63 (m, 1 H, Ar). Anal. Calcd for C_16_H_14_FN_3_OS_2_: C, 55.31; H, 4.06; N, 12.09. Found: C, 55.56; H, 3.79; N, 11.84.

#### 4.1.14. 3-(3-Fluorobenzyl)-2-((1-(ferrocen-2-yl)ethylidene)hydrazono)thiazolidin-4-one (15)

Brown oil, 69% yield; ^1 ^H NMR (400 MHz, DMSO-d_6_): *δ* 2.19 (s, 3H, CH_3_), 4.01 (s, 2H, CH_2_, thiazolidinone), 4.17 (s, 5H, ferrocene), 4.42 (s, 2H, ferrocene), 4.68 (s, 2H, ferrocene), 4.92 (s, 2 H, ArCH_2_), 7.12–7.23 (m, 3 H, Ar), 7.40–7.42 (m, 1H, Ar). ^13^C NMR (101 MHz, DMSO-d_6_) *δ* 16.2 (CH_3_), 32.5 (CH_2_, thiazolidinone), 45.8 (CH_2_), 67.8 (CH_2_, ferrocene), 69.7 (CH_2_, ferrocene), 70.6 (CH, ferrocene), 82.9 (C, ferrocene), 114.8 (Ar), 115.2 (Ar), 124.4 (Ar), 130.9 (Ar), 139.4 (Ar), 159.7 (C = N, thiazolidinone), 162.5 (d, *J_C–F_* = 244.3 Hz, Ar, C–F), 164.4 (C = N), 172.7 (C = O). Anal. Calcd for C_22_H_20_FFeN_3_OS: C, 58.81; H, 4.49; N, 9.35. Found: C, 58.99; H, 4.65; N, 9.51.

#### 4.1.15. 3–(4-Fluorobenzyl)-2-((heptan-3-ylidene)hydrazono)thiazolidin-4-one (16)

Yellow oil, 67% yield; ^1^H NMR (400 MHz, DMSO-d_6_): *δ* 0.78–0.81 (m, 1H, CH_2_), 0.86–0.90 (t, 3H, CH_3_), 1.02–1.05 (m, 2H, CH_2_), 1.12–1.16 (m, 1H, CH_2_), 1.25–1.34 (m, 2H, CH_2_), 1.47–1.51 (m, 1H, CH_2_), 2.24–2.34 (m, 4H, CH_2_ + CH_3_), 3.97 (s, 2H, CH_2_, thiazolidinone), 4.83 (s, 2 H, ArCH_2_), 7.12–7.18 (m, 2H, Ar), 7.34–7.38 (m, 2H, Ar). ^13^C NMR (101 MHz, DMSO-d_6_) *δ* 11.0 (CH_3_), 14.1 (CH_3_), 22.8 (CH_2_), 28.6 (CH_2_), 29.6 (CH_2_), 31.0 (CH_2_), 32.4 (CH_2_, thiazolidinone), 45.5 (CH_2_), 115.4 (Ar), 115.7 (Ar),130.1 (Ar), 130.2 (Ar) 132.9 (Ar), 160.4 (C = N, thiazolidinone), 160.8 (Ar, C–F), 163.2 (C = N), 172.6 (C = O). Anal. Calcd for C_17_H_22_FN_3_OS: C, 60.87; H, 6.61; N, 12.53. Found: C, 61.02; H, 6.77; N, 12.70.

#### 4.1.16. 3-(4-Fluorobenzyl)-2-((1-(thiophen-2-yl)ethylidene)hydrazono)thiazolidin-4-one (17)

Pink powder, mp 118–123 °C, 80% yield; ^1^H NMR (400 MHz, CDCl_3_): *δ* 2.47 (s, 3H, CH_3_), 3.81 (s, 2 H, CH_2_, thiazolidinone), 5.00 (s, 2H, ArCH_2_), 6.93–7.08 (m, 3H, thiophene), 7.22–7.52 (m, 4H, Ar). Anal. Calcd for C_16_H_14_FN_3_OS_2_: C, 55.31; H, 4.06; N, 12.09. Found: C, 55.06; H, 3.91; N, 11.84.

#### 4.1.17. 3-(4-Fluorobenzyl)-2-((1-(ferrocen-2-yl)ethylidene)hydrazono)thiazolidin-4-one (18)

Red powder, mp 122–127 °C, 82% yield; ^1^H NMR (400 MHz, DMSO-d_6_): *δ* 2.22 (s, 3H, CH_3_), 3.98 (s, 2H, CH_2_, thiazolidinone), 4.17 (s, 5H, ferrocene), 4.42 (s, 2H, ferrocene), 4.69 (s, 2H, ferrocene), 4.89 (s, 2H, ArCH_2_), 7.17–7.21 (m, 2H, Ar), 7.43–7.462 (m, 2H, Ar). ^13^C NMR (101 MHz, DMSO-d_6_) *δ* 16.2 (CH_3_), 32.5 (CH_2_, thiazolidinone), 45.6 (CH_2_), 67.8 (CH_2_, ferrocene), 69.7 (CH_2_, ferrocene), 70.6 (CH, ferrocene), 83.0 (C, ferrocene), 115.5 (Ar), 115.7 (Ar), 130.7 (Ar), 130.8 (Ar), 132.9 (Ar), 159.8 (C = N, thiazolidinone), 162.1 (d, *J_C–F_* = 244.4 Hz, Ar, C–F), 164.4 (C = N), 172.6 (C = O). Anal. Calcd for C_22_H_20_FFeN_3_OS: C, 58.81; H, 4.49; N, 9.35. Found: C, 59.04; H, 4.71; N, 9.47.

#### 4.1.18. 3-(2-Chlorobenzyl)-2-((heptan-3-ylidene)hydrazono)thiazolidin-4-one (19)

Orange oil, 60% yield; ^1^H NMR (400 MHz, DMSO-d_6_): *δ* 0.66–0.76 (m, 3H, CH_3_), 0.85–0.90 (m, 1H, CH_2_), 1.00–1.05 (m, 2H, CH_2_), 1.12–1.20 (m, 1H, CH_2_), 1.25–1.32 (m, 2H, CH_2_), 1.43–1.48 (m, 1H, CH_2_), 2.16–2.26 (m, 4H, CH_2_ + CH_3_), 4.05 (s, 2H, CH_2_, thiazolidinone), 4.93 (s, 2H, ArCH_2_), 7.13–7.20 (m, 1H, Ar), 7.27–7.31 (m, 2H, Ar), 7.45–7.48 (m, 1H, Ar). ^13^C NMR (101 MHz, CDCl_3_) *δ* 10.6 (CH_3_), 13.8 (CH_3_), 22.8 (CH_2_), 28.7 (CH_2_), 30.0 (CH_2_), 31.2 (CH_2_), 32.4 (CH_2_, thiazolidinone), 44.2 (CH_2_), 126.8 (Ar), 127.6 (Ar), 128.1 (Ar), 128.5 (Ar), 129.5 (Ar), 132.9 (Ar), 156.9 (C = N, thiazolidinone), 158.7 (C = N), 171.9 (C = O). Anal. Calcd for C_17_H_22_ClN_3_OS: C, 58.02; H, 6.30; N, 11.94. Found: C, 58.14; H, 6.11; N, 11.76.

#### 4.1.19. 3-(2-Chlorobenzyl)-2-((1-(thiophen-2-yl)ethylidene)hydrazono)thiazolidin-4-one (20)

Pink powder, mp 100–102 °C, 87% yield; ^1^H NMR (400 MHz, CDCl_3_): *δ* 2.31 (s, 3H, CH_3_), 3.90 (s, 2H, CH_2_, thiazolidinone), 5.18 (s, 2H, ArCH_2_), 7.05–7.06 (m, 1H, thiophene), 7.22 (bs, 3H: 2 H thiophene +1H Ar), 7.36–7.41 (m, 3H, Ar). Anal. Calcd for C_16_H_14_ClN_3_OS_2_: C, 52.81; H, 3.88; N, 11.55. Found: C, 52.66; H, 3.63; N, 11.24.

#### 4.1.20. 3-(2-Chlorobenzyl)-2-((1-(ferrocen-2-yl)ethylidene)hydrazono)thiazolidin-4-one (21)

Red powder, mp 143–148 °C, 83% yield; ^1^H NMR (400 MHz, DMSO-d_6_): *δ* 2.08 (s, 3H, CH_3_), 4.06 (s, 2H, CH_2_, thiazolidinone), 4.13 (s, 5H, ferrocene), 4.41 (s, 2H, ferrocene), 4.66 (s, 2H, ferrocene), 5.00 (s, 2H, ArCH_2_), 7.24 (bs, 1H, Ar), 7.33 (bs, 2H, Ar), 7.50 (bs, 1H, Ar). ^13^C NMR (101 MHz, DMSO-d_6_) *δ* 16.0 (CH_3_), 32.6 (CH_2_, thiazolidinone), 44.0 (CH_2_), 67.8 (CH_2_, ferrocene), 69.7 (CH_2_, ferrocene), 70.5 (CH, ferrocene), 82.9 (C, ferrocene), 127.7 (Ar), 128.5 (Ar), 129.5 (Ar), 129.8 (Ar), 132.3 (Ar), 133.5 (Ar), 159.4 (C = N, thiazolidinone), 164.4 (C = N), 172.6 (C = O). Anal. Calcd for C_22_H_20_ClFeN_3_OS: C, 56.73; H, 4.33; N, 9.02. Found: C, 56.55; H, 4.15; N, 9.21.

#### 4.1.21. 3-(3-Chlorobenzyl)-2-((heptan-3-ylidene)hydrazono)thiazolidin-4-one (22)

Yellow oil, 68% yield; ^1^H NMR (400 MHz, DMSO-d_6_): *δ* 0.77–0.81 (m, 2H, CH_2_), 0.86–0.90 (m, 3H, CH_3_), 1.02–1.06 (m, 2H, CH_2_), 1.21–1.26 (m, 2H, CH_2_), 1.48–1.52 (m, 1H, CH_2_), 2.24–2.35 (m, 4H, CH_2_ + CH_3_), 3.98 (s, 2H, CH_2_, thiazolidinone), 4.85 (s, 2H, ArCH_2_), 7.28–7.40 (m, 4H, Ar). ^13^C NMR (101 MHz, DMSO-d_6_) *δ* 11.0 (CH_3_), 14.1 (CH_3_), 22.8 (CH_2_), 28.6 (CH_2_), 29.6 (CH_2_), 30.9 (CH_2_), 32.4 (CH_2_, thiazolidinone), 45.7 (CH_2_), 126.7 (Ar), 127.9 (Ar), 130.7 (Ar), 133.5 (Ar), 139.1 (Ar), 139.2 (Ar), 158.9 (C = N, thiazolidinone), 160.4 (C = N), 172.7 (C = O). Anal. Calcd for C_17_H_22_ClN_3_OS: C, 58.02; H, 6.30; N, 11.94. Found: C, 58.28; H, 6.08; N, 11.81.

#### 4.1.22. 3-(3-Chlorobenzyl)-2-((1-(thiophen-2-yl)ethylidene)hydrazono)thiazolidin-4-one (23)

Pink powder, mp 129–131 °C, 89% yield; ^1^H NMR (400 MHz, CDCl_3_): *δ* 2.26 (s, 3H, CH_3_), 3.62 (s, 2H, CH_2_, thiazolidinone), 4.78 (s, 2H, ArCH_2_), 6.85–6.88 (m, 1H, thiophene), 7.05–7.08 (m, 3 H, thiophene + Ar), 7.17–7.20 (m, 2H, Ar), 7.31 (s, 1H, Ar). Anal. Calcd for C_16_H_14_ClN_3_OS_2_: C, 52.81; H, 3.88; N, 11.55. Found: C, 52.99; H, 3.69; N, 11.73.

#### 4.1.23. 3-(3-Chlorobenzyl)-2-((1-(ferrocen-2-yl)ethylidene)hydrazono)thiazolidin-4-one (24)

Red powder, mp 143–144 °C, 87% yield; ^1^H NMR (400 MHz, DMSO-d_6_): *δ* 2.21 (s, 3 H, CH_3_), 4.01 (s, 2 H, CH_2_, thiazolidinone), 4.17 (s, 5 H, ferrocene), 4.43 (s, 2 H, ferrocene), 4.69 (s, 2 H, ferrocene), 4.90 (s, 2 H, ArCH_2_), 7.36–7.40 (m, 3 H, Ar), 7.47 (s, 1 H, Ar). ^13^C NMR (101 MHz, DMSO-d_6_) *δ* 16.2 (CH_3_), 32.5 (CH_2_, thiazolidinone), 45.7 (CH_2_), 67.8 (CH_2_, ferrocene), 69.7 (CH_2_, ferrocene), 70.6 (CH, ferrocene), 82.9 (C, ferrocene), 127.2 (Ar), 128.0 (Ar), 128.5 (Ar), 130.8 (Ar), 133.4 (Ar), 139.1 (Ar), 159.7 (C = N, thiazolidinone), 164.5 (C = N), 172.7 (C = O). Anal. Calcd for C_22_H_20_ClFeN_3_OS: C, 56.73; H, 4.33; N, 9.02. Found: C, 56.90 H, 4.04; N, 9.18.

#### 4.1.24. 3-(4-Chlorobenzyl)-2-((heptan-3-ylidene)hydrazono)thiazolidin-4-one (25)

White oil, 60% yield; ^1^H NMR (400 MHz, DMSO-d_6_): *δ* 0.54–0.57 (m, 2H, CH_2_), 0.62–0.70 (m, 3H, CH_3_), 0.81–0.84 (q, 2H, CH_2_), 0.89–0.93 (m, 1H, CH_2_), 1.08–1.13 (m, 1H, CH_2_), 1.27–1.30 (m, 1H, CH_2_), 2.03–2.11 (m, 4H, CH_2_ + CH_3_), 3.78 (s, 2H, CH_2_, thiazolidinone), 4.63 (s, 2H, ArCH_2_), 7.11–7.19 (m, 4H, Ar). ^13^C NMR (101 MHz, DMSO-d_6_) *δ* 11.0 (CH_3_), 14.1 (CH_3_), 22.8 (CH_2_), 28.6 (CH_2_), 29.6 (CH_2_), 31.0 (CH_2_), 32.4 (CH_2_, thiazolidinone), 45.6 (CH_2_), 128.8 (Ar), 129.8 (Ar), 132.5 (Ar), 135.7 (Ar), 158.8 (C = N, thiazolidinone), 160.2 (C = N), 172.6 (C = O). Anal. Calcd for C_17_H_22_ClN_3_OS: C, 58.02; H, 6.30; N, 11.94. Found: C, 57.81; H, 6.64; N, 12.16.

#### 4.1.25. 3-(4-Chlorobenzyl)-2-((1-(thiophen-2-yl)ethylidene)hydrazono)thiazolidin-4-one (26)

White powder, mp 146–151 °C, 81% yield; ^1^H NMR (400 MHz, DMSO-d_6_): *δ* 2.34 (s, 3 H, CH_3_), 4.03 (s, 2 H, CH_2_, thiazolidinone), 4.90 (s, 2 H, ArCH_2_), 7.12 (bs, 1 H, thiophene), 7.41 (bs, 4 H, Ar), 7.54 (bs, 1 H, thiophene), 7.61–7.62 (m, 1 H, thiophene). Anal. Calcd for C_16_H_14_ClN_3_OS_2_: C, 52.81; H, 3.88; N, 11.55. Found: C, 53.05; H, 4.02; N, 11.32.

#### 4.1.26. 3-(4-Chlorobenzyl)-2-((1-(ferrocen-2-yl)ethylidene)hydrazono)thiazolidin-4-one (27)

Red powder, mp 167–169 °C, 87% yield; 1^ ^H NMR (400 MHz, DMSO-d_6_): *δ* 2.21 (s, 3H, CH_3_), 3.99 (s, 2H, CH_2_, thiazolidinone), 4.17 (s, 5H, ferrocene), 4.42 (s, 2H, ferrocene), 4.68 (s, 2H, ferrocene), 4.89 (s, 2H, ArCH_2_), 7.42 (bs, 4H, Ar). ^13^C NMR (101 MHz, DMSO-d_6_) *δ* 16.2 (CH_3_), 32.5 (CH_2_, thiazolidinone), 45.6 (CH_2_), 67.8 (CH_2_, ferrocene), 69.7 (CH_2_, ferrocene), 70.6 (CH, ferrocene), 82.9 (C, ferrocene), 128.8 (Ar), 130.4 (Ar), 132.7 (Ar), 135.7 (Ar), 159.8 (C = N, thiazolidinone), 164.4 (C = N), 172.6 (C = O). Anal. Calcd for C_22_H_20_ClFeN_3_OS: C, 56.73; H, 4.33; N, 9.02. Found: C, 56.95 H, 4.57; N, 8.83.

#### 4.1.27. 2-((Heptan-3-ylidene)hydrazono)-3-(naphthalen-1-ylmethyl)thiazolidin-4-one (28)

Orange oil, 65% yield; ^1^H NMR (400 MHz, CDCl_3_): *δ* 0.83–0.87 (t, 3 H, CH_3_), 0.92–0.96 (t, 3 H, CH_3_), 1.23–1.37 (m, 5 H, CH_2_), 2.26–2.34 (m, 3 H, CH_2_), 3.84 (s, 2 H, CH_2_, thiazolidinone), 5.46 (s, 2 H, ArCH_2_), 7.40–7.58 (m, 4 H, Ar), 7.80–7.92 (m, 2 H, Ar), 8.21–8.27 (m, 1 H, Ar). Anal. Calcd for C_21_H_25_N_3_OS: C, 68.63; H, 6.86; N, 11.43. Found: C, 68.81; H, 6.99; N, 11.60.

#### 4.1.28. 3-(Naphthalen-1-ylmethyl)-2-((1-(ferrocen-2-yl)ethylidene)hydrazono)thiazolidin-4-one (30)

Red oil, 63% yield; 1^ ^H NMR (400 MHz, DMSO-d_6_): *δ* 2.08 (s, 3 H, CH_3_), 4.07 (s, 2 H, CH_2_, thiazolidinone), 4.15 (s, 5 H, ferrocene), 4.41 (s, 2 H, ferrocene), 4.66 (s, 2 H, ferrocene), 5.38 (s, 2 H, ArCH_2_), 7.43–7.62 (m, 4 H, Ar), 7.88–7.90 (m, 1 H, Ar), 7.98–7.99 (m, 1 H, Ar), 8.31–8.33 (m, 1 H, Ar). Anal. Calcd for C_26_H_23_FeN_3_OS: C, 64.87; H, 4.82; N, 8.73. Found: C, 64.59; H, 4.64; N, 8.91.

#### 4.1.29. 2-((2-((Heptan-3-ylidene)hydrazono)-4-oxothiazolidin-3-yl)methyl)isoindoline-1,3-dione (31)

Yellow oil, 86% yield; 1^ ^H NMR (400 MHz, CDCl_3_): *δ* 0.82–0.93 (m, 2 H, CH_2_, 3 H, CH_3_), 1.08–1.12 (m, 1H, CH_2_), 1.30–1.37 (m, 3H, CH_3_), 1.51–1.55 (m, 1H, CH_2_), 2.24–2.32 (m, 2H, CH_2_), 2.36–2.44 (m, 2H, CH_2_), 3.82 (s, 2H, CH_2_, thiazolidinone), 5.71 (s, 2H, ArCH_2_), 7.73–75 (m, 2H, Ar), 7.85–7.86 (m, 2H, Ar). Anal. Calcd for C_19_H_22_N_4_O_3_S: C, 59.05; H, 5.74; N, 14.50. Found: C, 59.31; H, 5.99; N, 14.21.

#### 4.1.30. 2-((4-Oxo-2-((1-(ferrocen-2-yl)ethylidene)hydrazono)thiazolidin-3-yl)methyl)isoindoline-1,3-dione (33)

Red powder, mp 235–237 °C, 91% yield; 1^ ^H NMR (400 MHz, DMSO-d_6_): *δ* 2.08 (s, 3H, CH_3_), 3.98 (s, 2H, CH_2_, thiazolidinone), 4.08 (s, 5H, ferrocene), 4.39 (s, 2H, ferrocene), 4.62 (s, 2H, ferrocene), 5.59 (s, 2H, ArCH_2_), 7.88–7.91 (m, 4H, Ar). Anal. Calcd for C_24_H_20_FeN_4_O_3_S: C, 57.61; H, 4.03; N, 11.20. Found: C, 57.34; H, 4.20; N, 11.05.

### 4.2. Five-day growth inhibition assay

Compounds were tested for the ability to inhibit *in vitro* tachyzoite (*T. gondii* RH-2 F (50839); ATCC, VA) and human foreskin fibroblast (HFF; ATCC) growth for 120 h using a published colorimetric assay^8^^,^^19^. Stock solutions of all compounds were 10 mM in DMSO. Serial 0.5 log_10_ dilutions (320–0.032 μM) of each compound were tested. Data from three separate experiments were analysed and the IC_50_, IC_90_, and TD_50_ calculated using Calcusyn software (Biosoft, Cambridge, UK). The therapeutic index (TI), a measure of specific anti-*T. gondii* activity, was calculated with the formula shown under Table 1.

### 4.3. Invasion assay

The ferrocene-based derivatives (10 μM) were examined for activity directly on extracellular tachyzoites using an established immunofluorescence-based red/green invasion assay^19^. This assay measures a compound’s ability to impede the parasite-driven host cell entry of tachyzoites through the host cell plasma membrane. Invaded (intracellular) tachyzoites are labelled green while tachyzoites attached to the surface, but unable to invade, are labelled red. A decrease in the number of invaded tachyzoites relative to vehicle [VHL (DMSO)] is indicative of invasion inhibition. A decrease in the number of invaded plus attached tachyzoites relative to same of VHL is indicative of inhibition of attachment. Data shown are mean values ± SEM of three independent experiments.

### 4.4. Replication assay

The ferrocene-based derivatives (10 μM) were also tested for inhibitory activity against intracellular tachyzoites that had been allowed to invade host cells and establish an infection for 2 h before the addition of compound using an established immunofluorescence-based protocol^19^. This assay measures a compound’s ability to inhibit tachyzoite replication over a period of 24 h. The numbers of parasitophorous vacuoles (PV) in experimentally treated infected cells containing 1, 2, 4, or 8 + tachyzoites/vacuole were enumerated, graphed, and compared to those of the VHL-treated infected cells. Data for each size vacuole are shown as the fraction of the total number of vacuoles counted in 10–15 fields. Data shown were compiled from three independent experiments. A decrease in the number of tachyzoites in a vacuole indicates inhibition of replication. *Toxoplasma gondii* tachyzoites only replicate within the PV inside the host cell with one cycle of replication taking 6–8 h. Therefore, PVs containing 1, 2, 4, or 8 tachyzoites indicates that 0, 1, 2, or 3 cycles of replication have been completed. Consequently, a decrease in the number of intravacuolar tachyzoites relative to that of VHL indicates inhibition of replication^21^. Specifically, predominance, i.e. the largest fraction of the total number, of PVs containing 1 or 2 tachyzoites indicates replication inhibition.

## 5. Results and discussion

We synthesised 33 new *N*-substituted thiazolidin-4-one derivatives in high yield keeping constant three substituents at the *N*1-hydrazine portion of the scaffold (3-heptanone, 2-acetylthiophene and acetylferrocene) from the good results obtained in our previous works^19^. We explored the chemical space and the influence on the biological activity of different groups (substituted aromatic and bicyclic rings) at the lactam NH of the core nucleus. We have confirmed that this thiazolidin-4-one scaffold can be as effective, or better than, the reference drug sulfadiazine *in vitro* against the tachyzoites of *T. gondii*. In the five-day growth inhibition assay, compounds were added only once, just before addition of tachyzoites, and thus were present for multiple cycles, up to 20, of parasite replication. Specifically, compounds were tested against, (i) the initial inoculum of tachyzoites as they attempt to invade host cells, (ii) intracellular tachyzoites attempting to replicate within the PV, and (iii) tachyzoites that have egressed from the initial infected cells and are moving out to infect neighbouring cells. Thus, compounds that were highly efficacious in this assay inhibited both host cell invasion and replication. Additionally, such compounds were stable at 37 °C and capable of being transported not only across the cell plasma membrane, but across the PV membrane. In this environment then, the best of the newly synthesised thiazolidinones, in terms of IC_50_ and TI, were all those deriving from acetylferrocene (**3**, **6, 12**, **15**, **18**, **21**, **24**, **27,** and **30**), showing better values than reference compound sulfadiazine (Table 1). The only exceptions were compounds **9** and **33**. In particular, in the ferrocene-containing derivatives it is possible to state that the substitution of the lactam NH influenced the biological activity with compound **30** as the best active and least cytotoxic derivative. Moreover, regarding the influence of the substituent and its position on the aromatic ring from Table 1 it can be extrapolated by analysing IC_50_ and TI data that 4-F (**18**) > 3-F (**15**) > 2-F (**12**), 3-Cl (**24**) ≥ 2-Cl (**21**) > 4-Cl (**27**), and 2-NO_2_ (**3**) > 3-NO_2_ (**6**) > 4-NO_2_ (**9**). Only two derivatives (**1**, **13**) in the 3-heptanone series displayed promising biological activity better than reference drug. In general, phthalimide-based derivatives (at the lactam NH) displayed minimal growth inhibitory activity.

We next evaluated the ability of the ferrocene-based derivatives to act directly upon extracellular tachyzoites by using a standard red/green invasion assay. This assay interrogates compounds for inhibition of the first step in host cell infection, i.e. invasion and entry into susceptible cells. In this assay (Figure 1), cell-free purified tachyzoites were incubated with a test compound (10 μM), sulfadiazine (10 μM), or VHL for 20 min at room temperature before being added to HFF host cells. After allowing 1 h at 37 °C for the tachyzoites to attach and invade, the cells were processed by immunofluorescent staining. Extracellular/attached tachyzoites were labelled red while intracellular/penetrated tachyzoites were labelled green. We found that all of the ferrocene compounds, with the notable exceptions of compounds **3** and **27**, significantly inhibited invasion. Additionally, compounds **9**, **15,** and **30** significantly inhibited attachment of tachyzoites to the HFF cells (Figure 1).

Lastly, the replication assay interrogates a compound’s ability to inhibit an established intracellular *Toxoplasma* infection over the relatively short period of 24 h (3–4 cycles of replication). In this assay, robust inhibition of replication results in PVs containing only one tachyzoite indicating that a treatment did not allow any replication to occur. Likewise, allowance of just one cycle of replication results in a majority of PVs containing just two tachyzoites. However, a predominance of PVs containing eight or more tachyzoites indicates absence of inhibitory activity over a 24 h period (see detailed explanation above in Experimental Protocols). As shown in Figure 2 the reference compound, sulfadiazine, showed robust replication inhibition during this short period with a predominance of PVs containing just one or two tachyzoites. Somewhat surprisingly, only one of the ferrocene derivatives, compound **27**, displayed fairly strong inhibition with the predominant number of PVs containing two tachyzoites and ≤1% containing eight or more. Treatment with derivatives **12** and **18** resulted in modest inhibition with a predominance of PVs containing only two tachyzoites but also 10–18% containing eight tachyzoites (Figure 2). In light of the efficacy displayed in the five-day growth inhibition and invasion assays, these results suggest that the majority of the ferrocene derivatives exert their anti-*Toxoplasma* activity primarily against the tachyzoite itself and are unable to affect intracellular tachyzoites. This could be due to inefficient transport across cell and PV membranes. This aspect will be investigated when designing new chemical modifications within this scaffold.

## 6. Conclusions

We have synthesised a large number of *N*-substituted 1,3-thiazolidin-4-one derivatives to assess their inhibitory activity against *Toxoplasma* tachyzoites. Approximately one-third of them stood out as promising antiparasitic agents possessing an activity similar or better than that of sulfadiazine against *Toxoplasma* and a comparable or lower cytotoxicity with respect to the reference drug. Furthermore, we have also documented that among the most active compounds, some derivatives strongly blocked the parasite attachment and invasion of the host cell. From the comparison of these data and the ones reported in our previous paper^19^, we can assess that the presence of a ferrocene-based thiazolidinone is crucial for the anti-*Toxoplasma* activity disregarding the type and nature of the substituent at the lactam NH. However, a strong improvement in this biological activity was observed by the introduction of substituted benzyl groups with respect to phthalimide or naphthalene ring at this position. These results demonstrated the promising potential of this thiazolidinone scaffold for the development of new anti-parasitic drugs.

## Supplementary Material

IENZ_1316494_Supplementary_Material.pdf
