# Supplementary material for: Synthesis and biological evaluation of anti-Toxoplasma gondii activity of a novel scaffold of thiazolidinone derivatives
Source: J Enzyme Inhib Med Chem. 2017 May 24;32(1):746–58. doi: 10.1080/14756366.2017.1316494 (PMC6445228; doi:10.1080/14756366.2017.1316494)

Supporting infomation

Synthesis and biological evaluation of anti-*Toxoplasma gondii* activity of a novel scaffold of thiazolidinone derivatives

Simone Carradori[a], Daniela Secci[b*], Bruna Bizzarri[b], Paola Chimenti[b], Celeste De Monte[b], Paolo Guglielmi[b], Cristina Campestre[a], Daniela Rivanera[c], Claudia Bordón[d], Lorraine Jones-Brando[d]

[a]*Department of Pharmacy, "G. D'Annunzio" University of Chieti-Pescara, Via dei Vestini 31, 66100, Chieti, Italy*

[b]*Department of Drug Chemistry and Technologies, Sapienza University of Rome, P.le A. Moro 5, 00185 Rome, Italy*

[c]*Department of Public Health and Infectious Diseases, Sapienza University of Rome, P.le A. Moro 5, 00185 Rome, Italy*

[d]*Stanley Division of Developmental Neurovirology, Johns Hopkins University School of Medicine, 600 North Wolfe Street, Blalock 1105, Baltimore, Maryland 21287, USA*

[*]Corresponding author: Prof. Daniela Secci: Tel/fax: +39 06 49913763; e-mail: daniela.secci@uniroma1.it

Figure S1. ^{13}C NMR spectrum of compound **6**

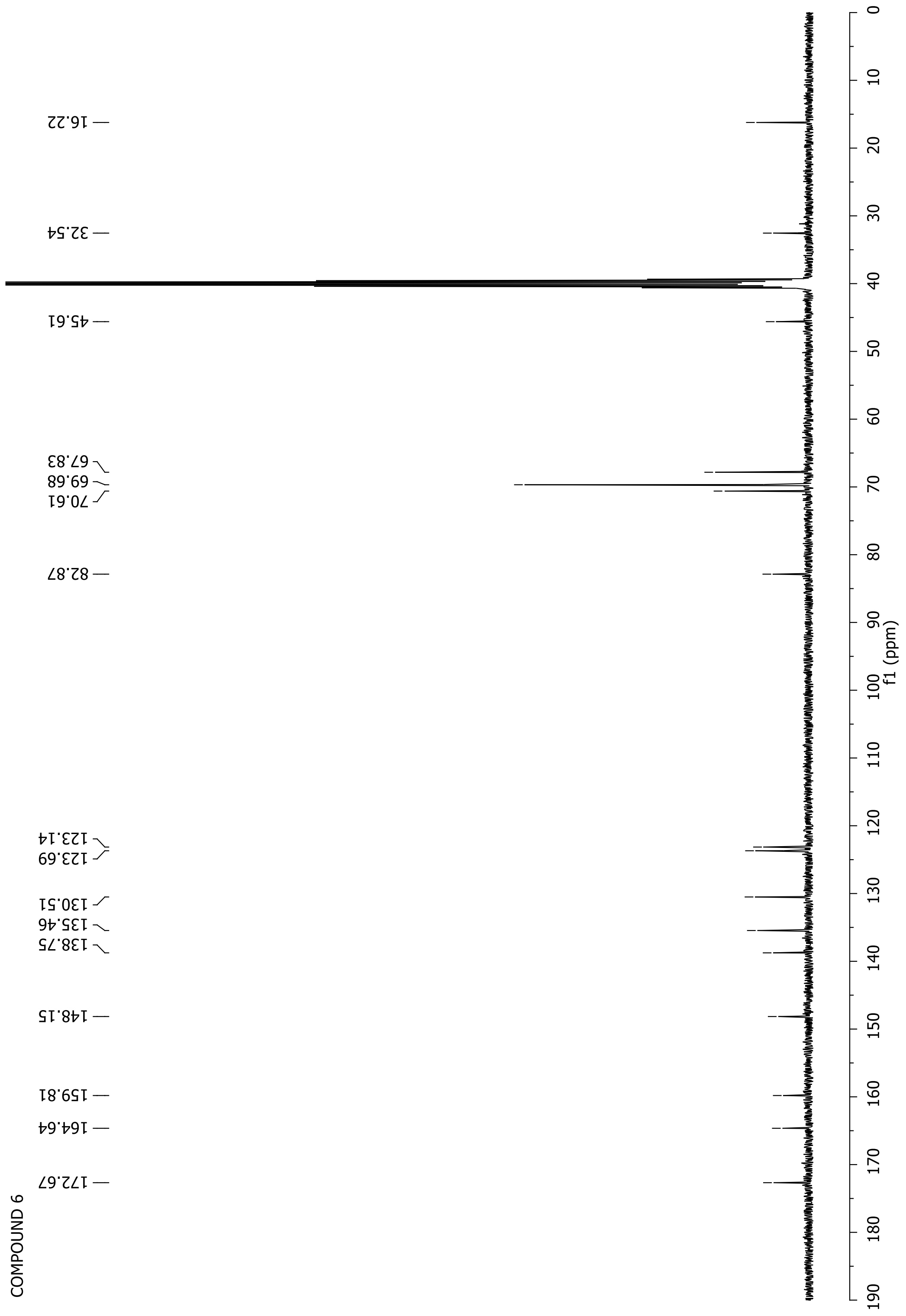

Figure S2. ^{13}C NMR spectrum of compound **9**

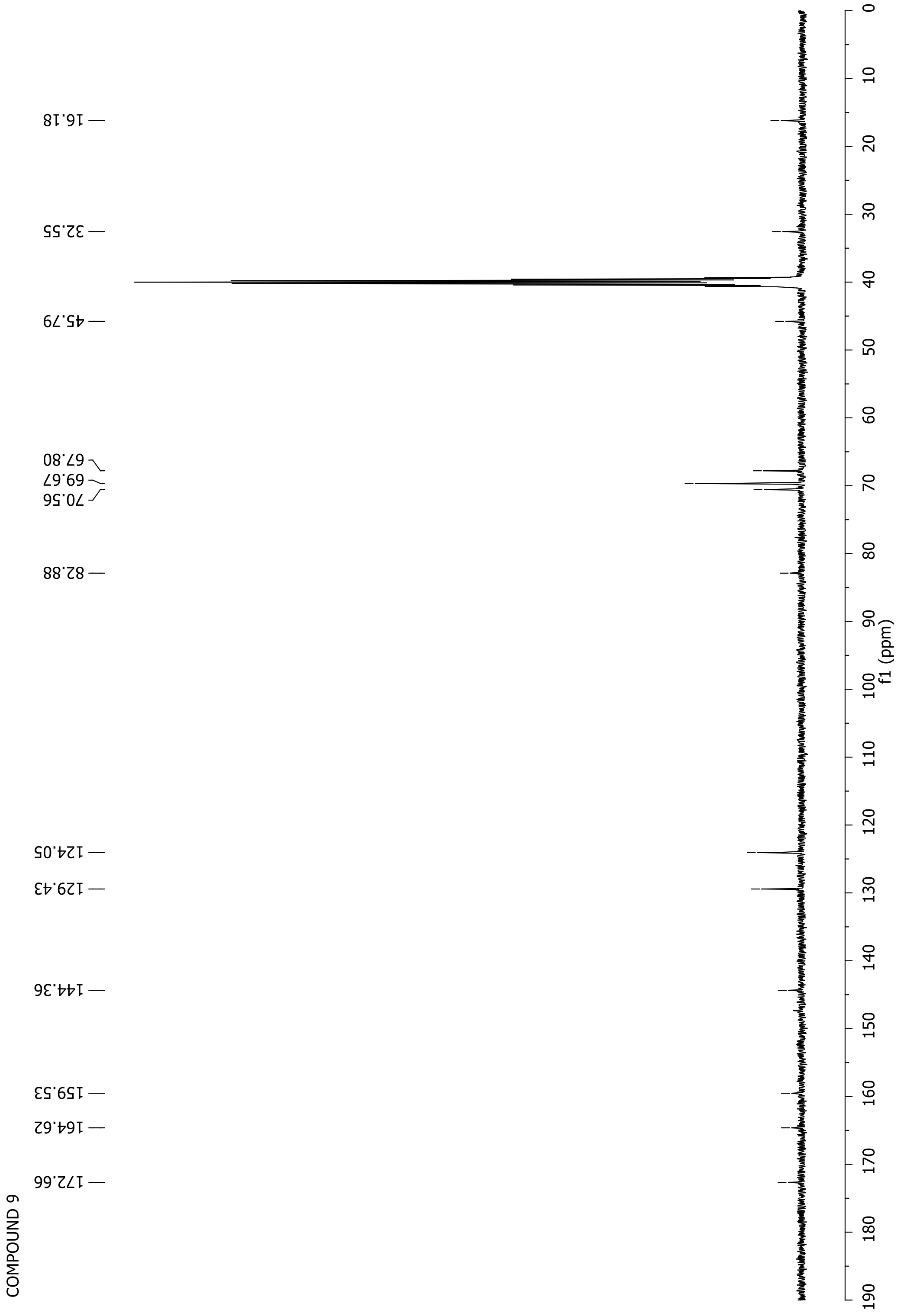

Figure S3. ^{13}C NMR spectrum of compound **15**

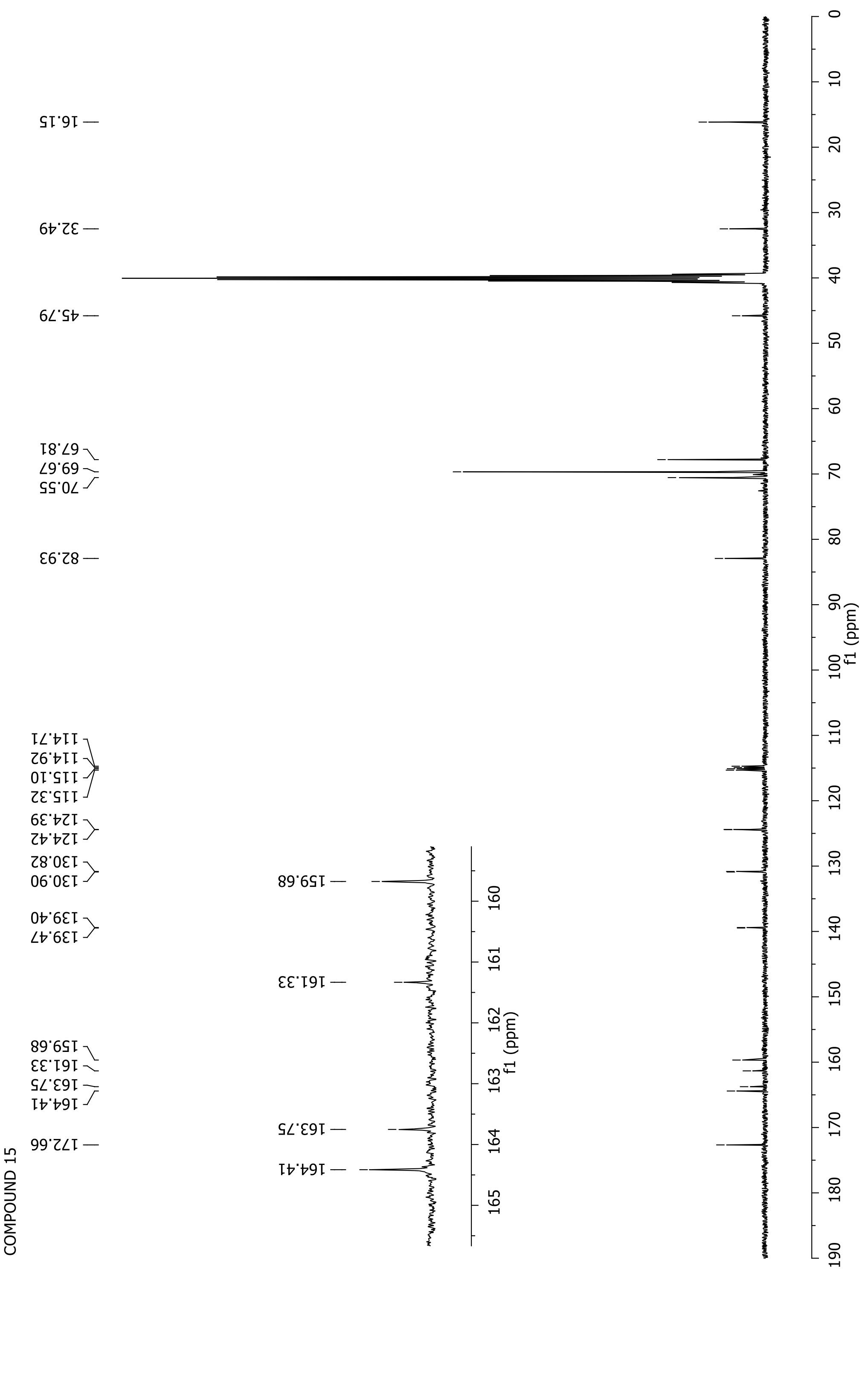

Figure S4. ^{1}H NMR spectrum of compound **18**

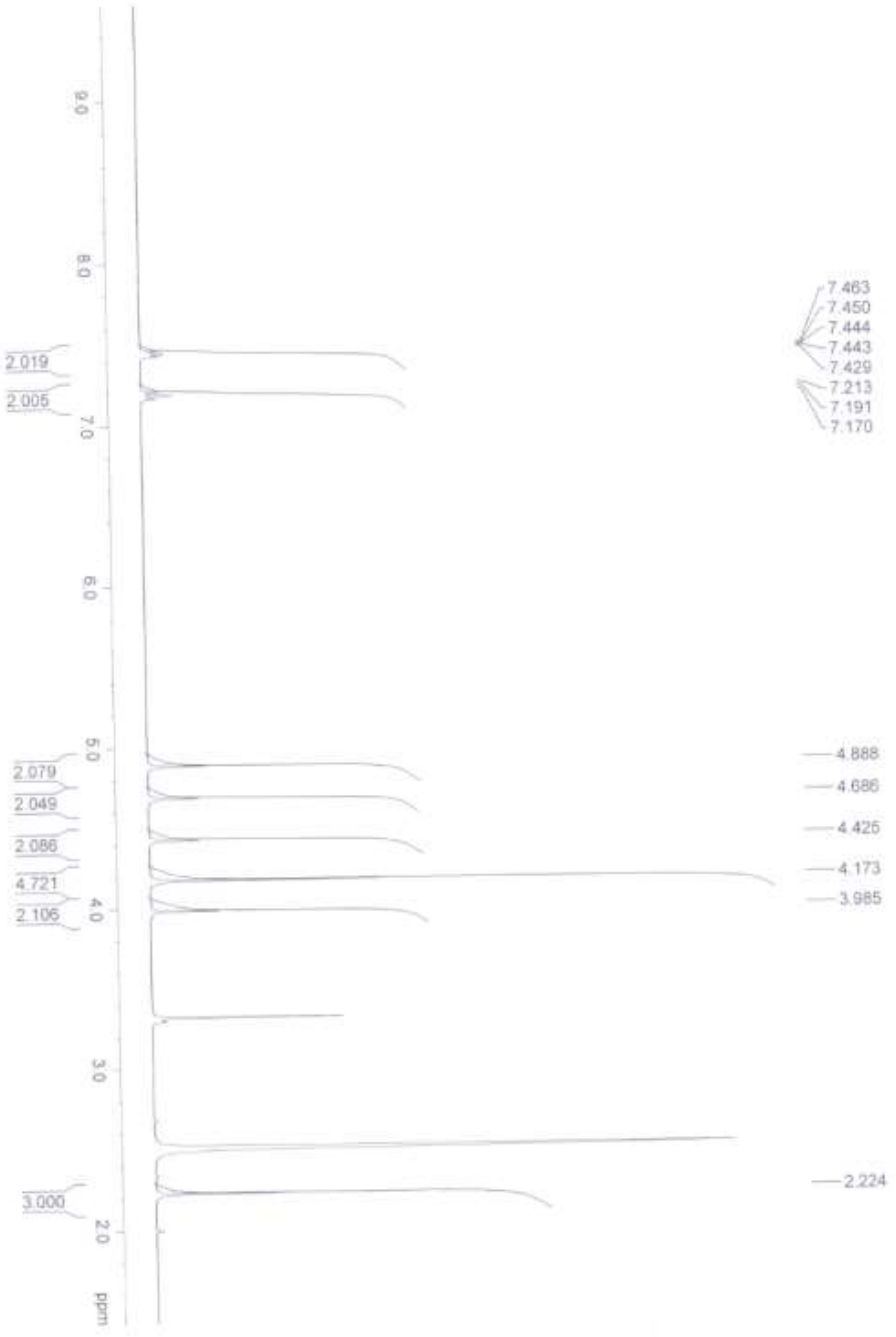

Figure S5. ^{13}C NMR spectrum of compound **18**

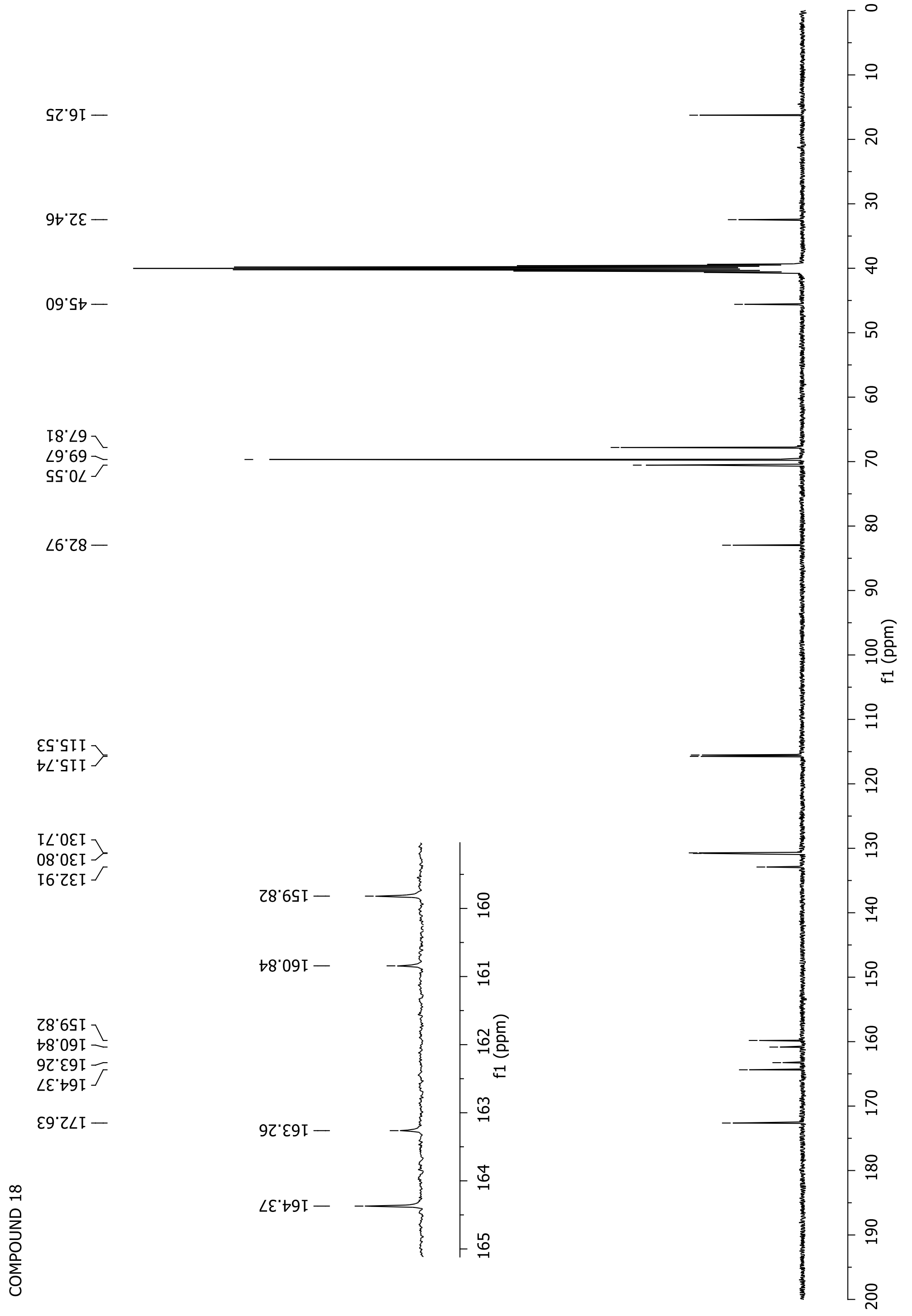

Figure S6. ^{1}H NMR spectrum of compound **21**

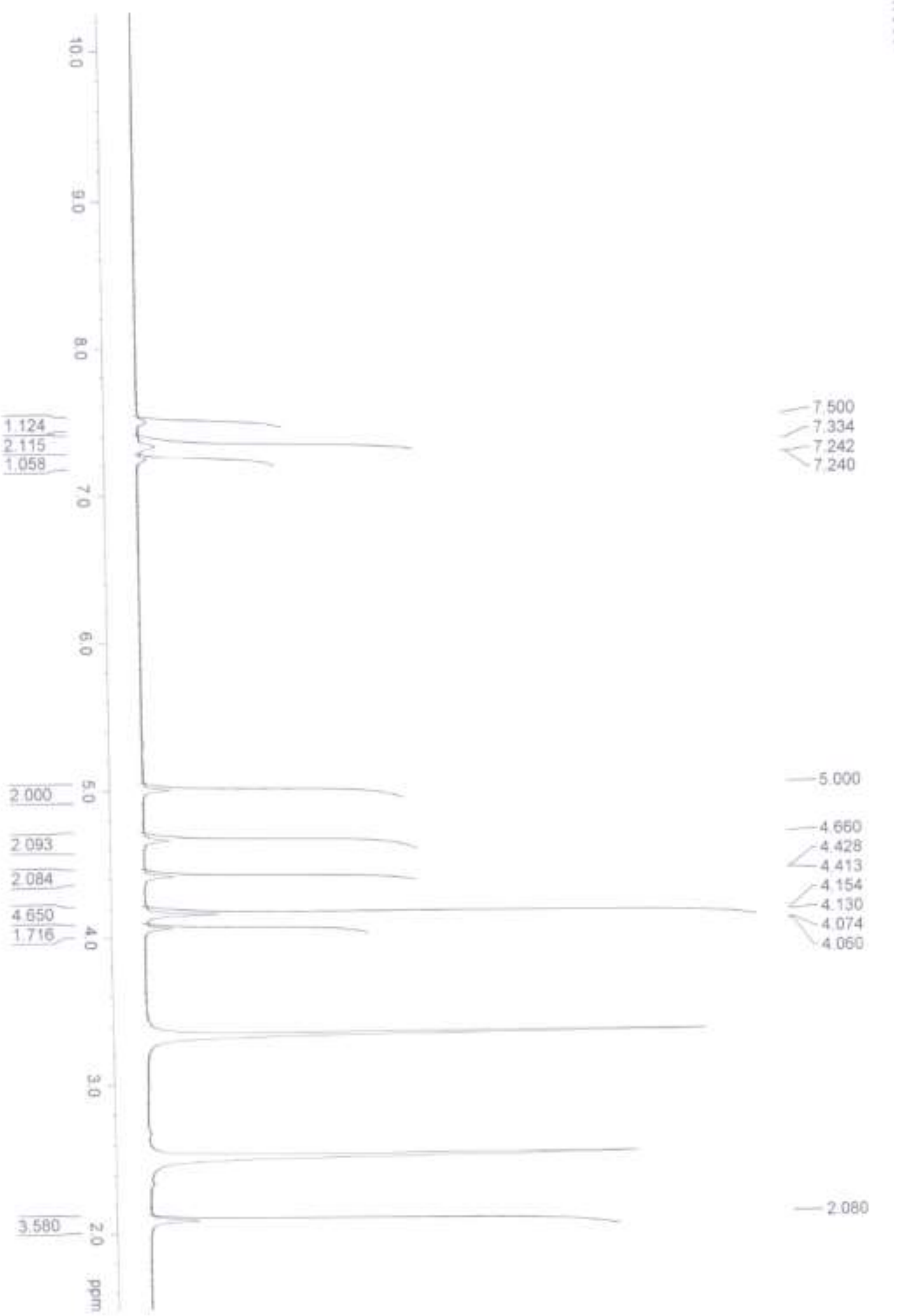

Figure S7. ^{13}C NMR spectrum of compound **21**

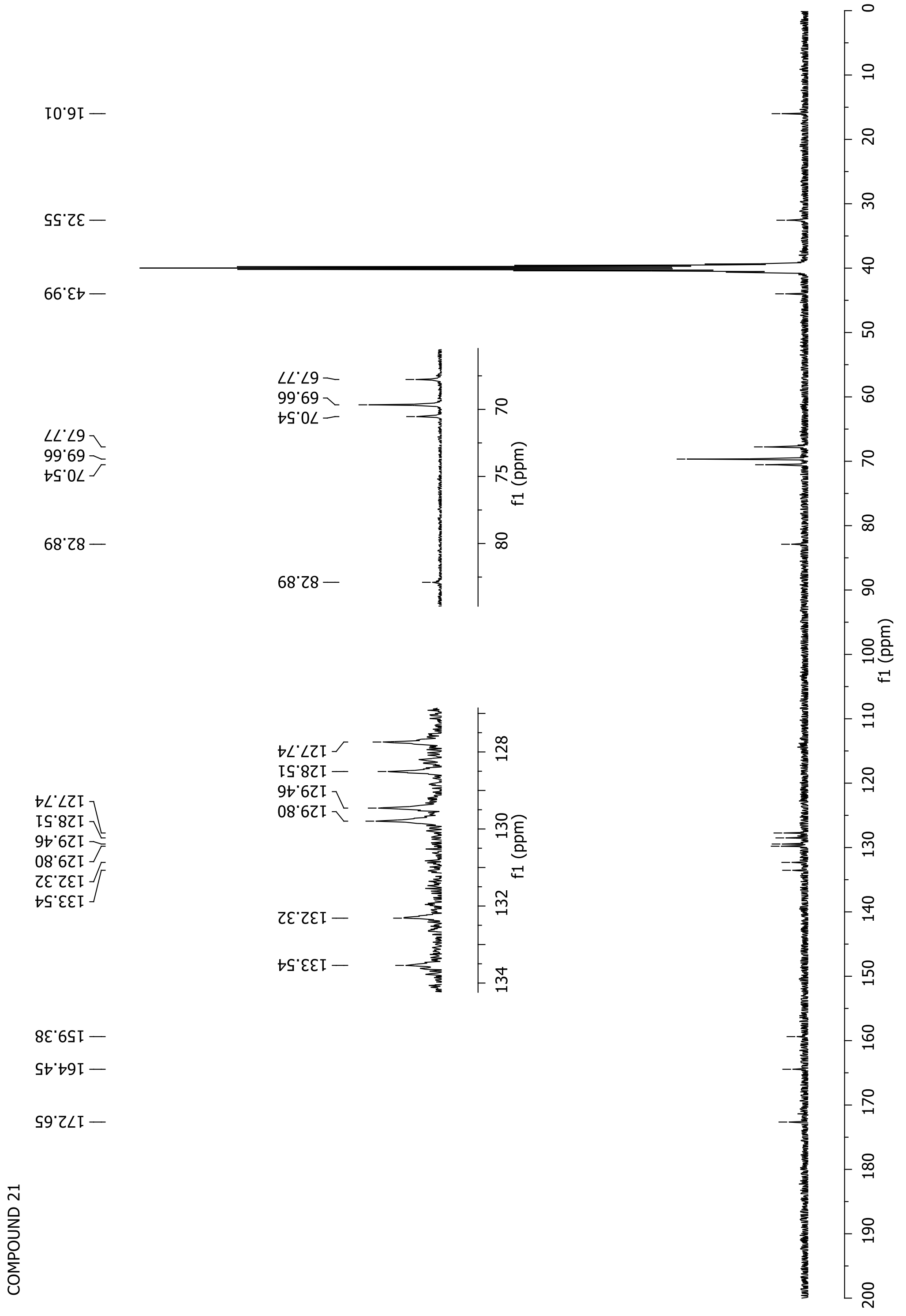

Figure S8. ^{1}H NMR spectrum of compound **24**

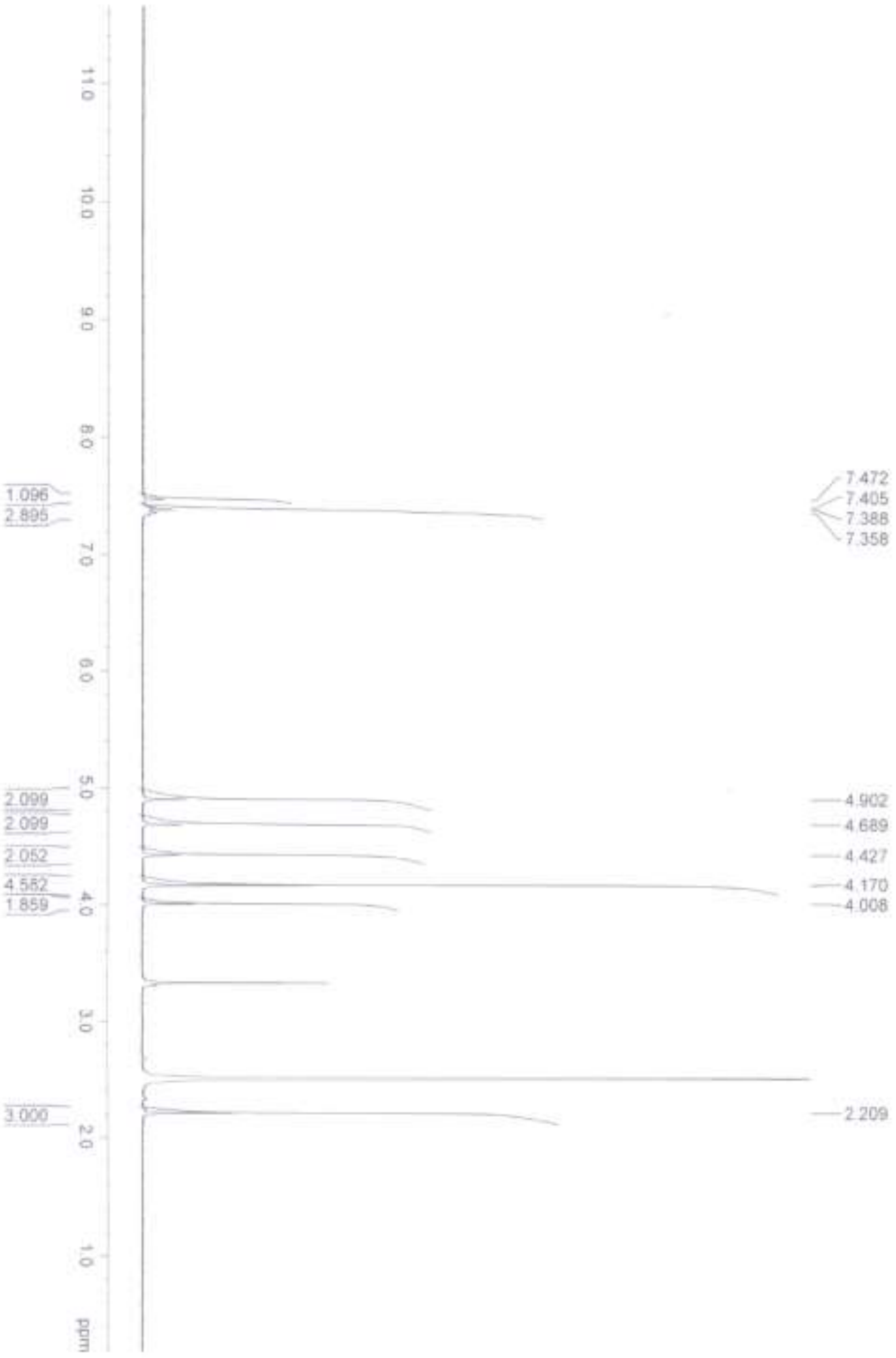

Figure S9. ^{13}C NMR spectrum of compound **24**

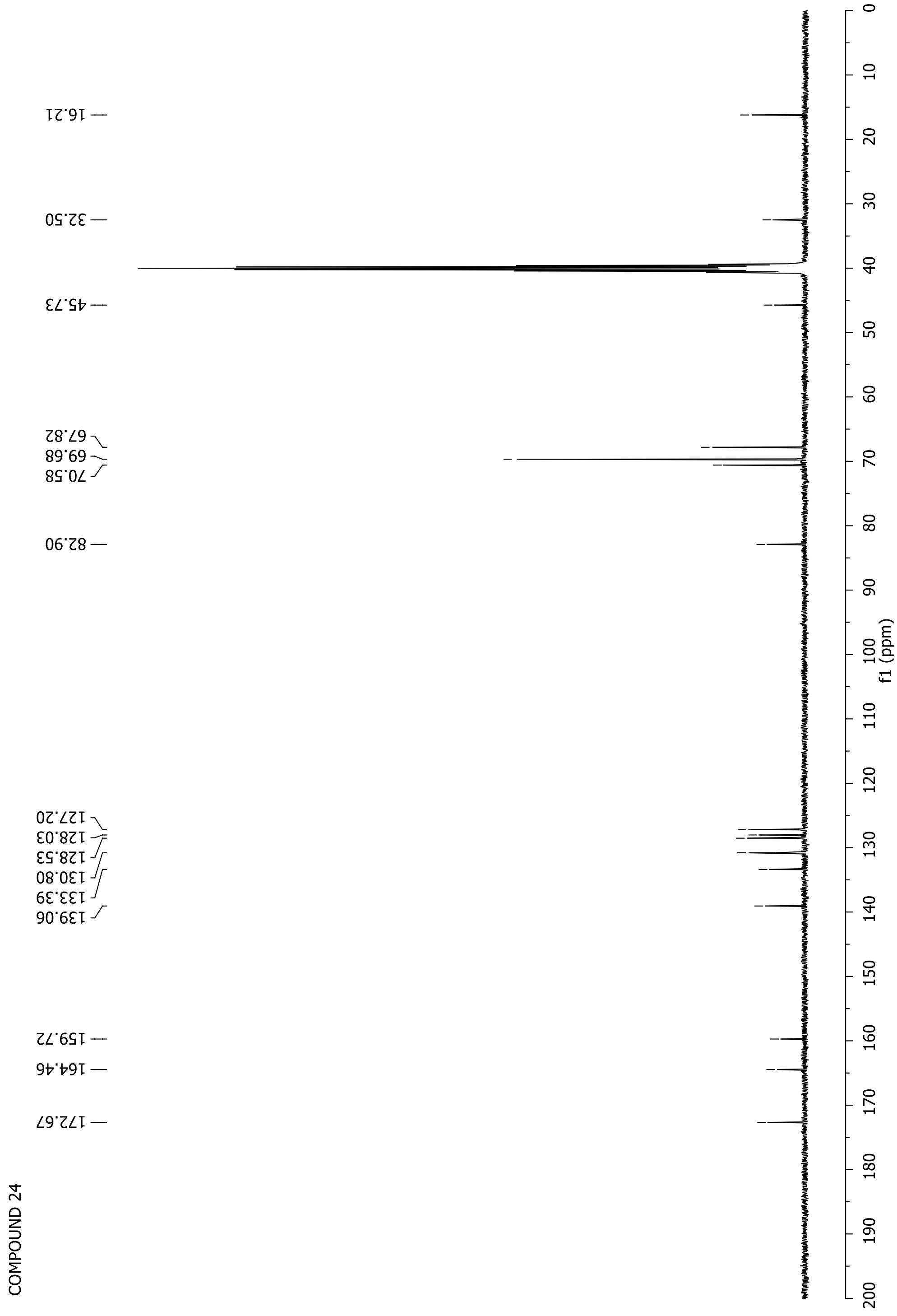

Figure S10. ^{1}H NMR spectrum of compound **27**

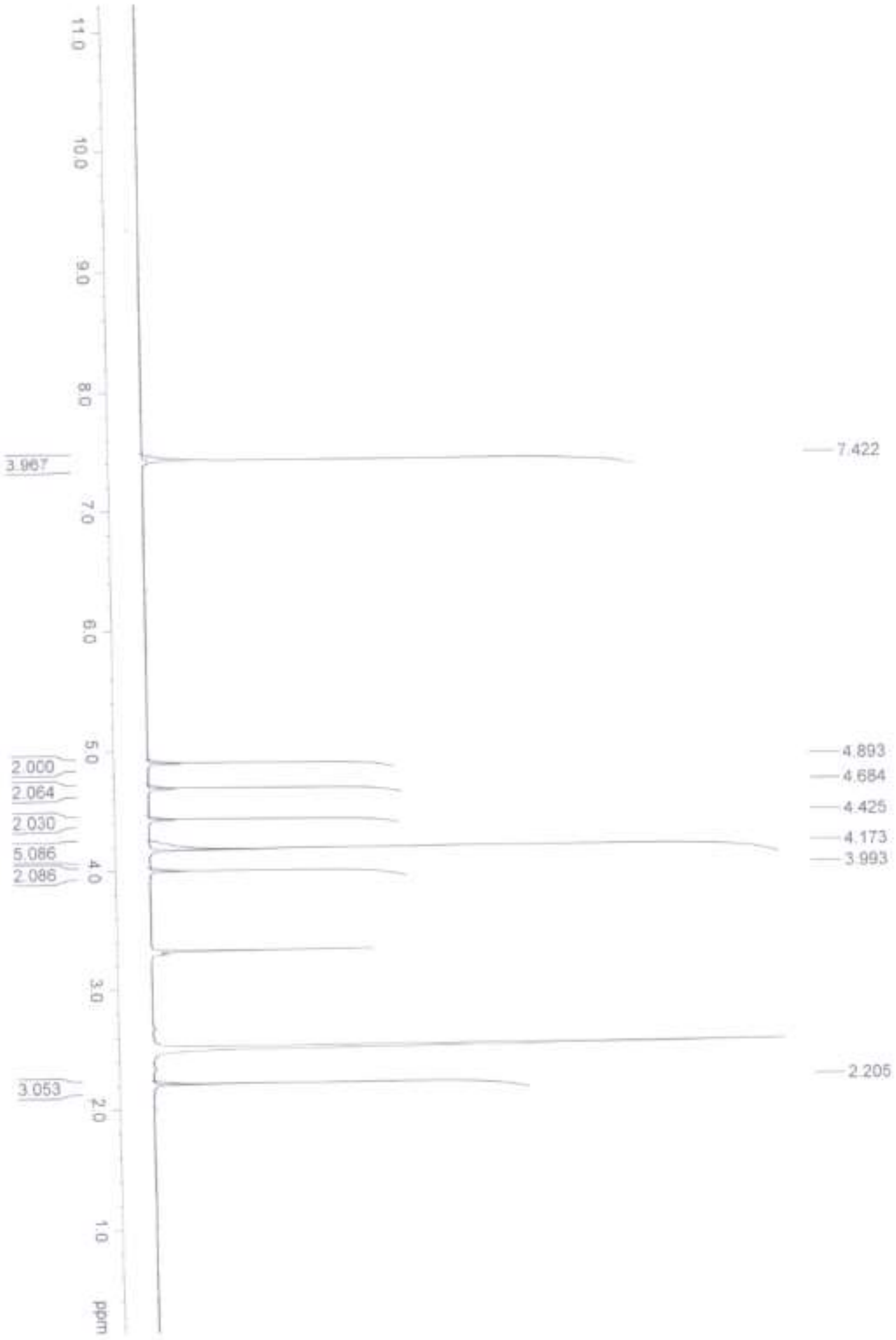

Figure S11. ^{13}C NMR spectrum of compound **27**

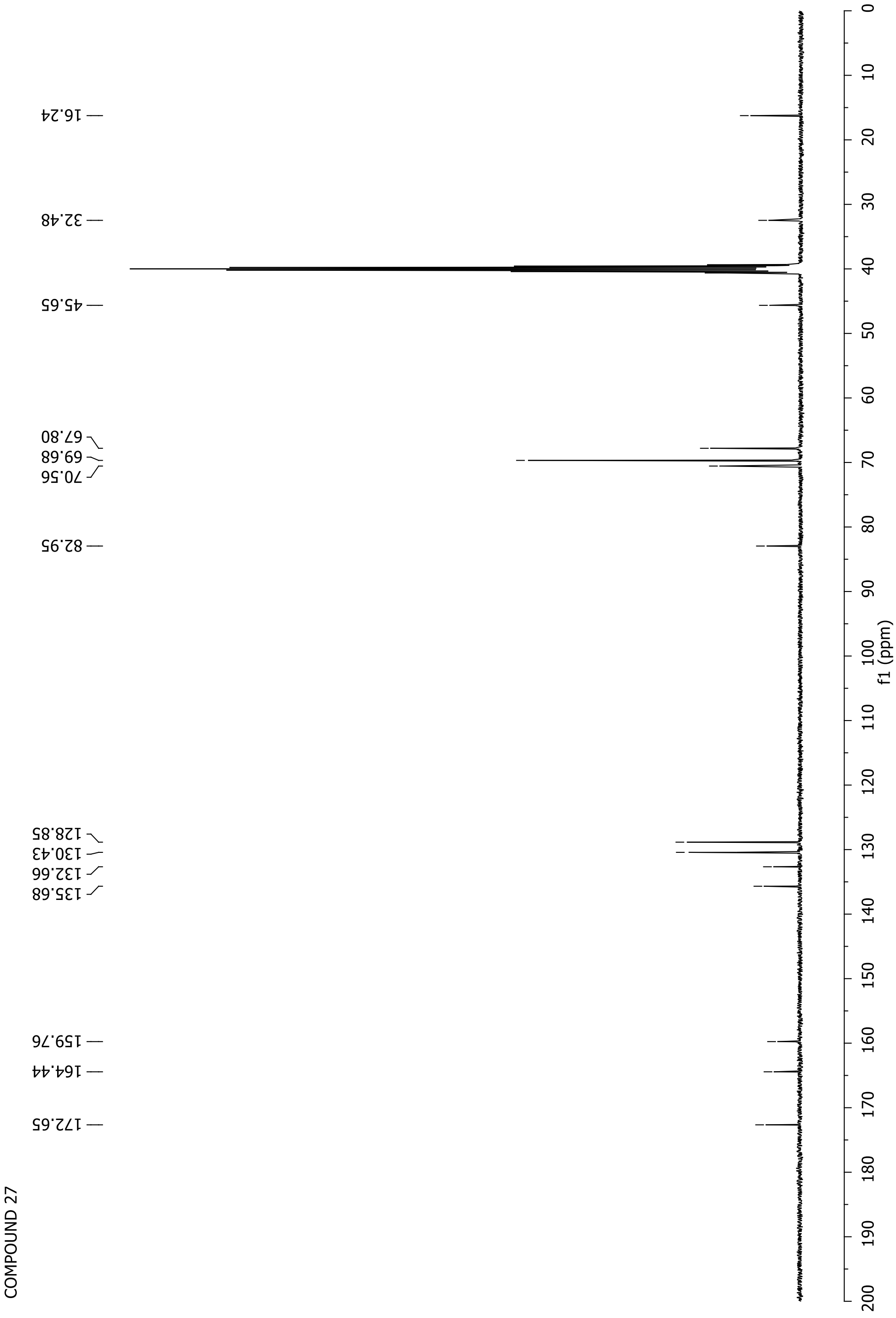

Supplement: IENZ_1316494_Supplementary_Material.pdf [file IENZ_A_1316494_SM3605.pdf]
